# Supplementary material for: Integrative phylogenomic and pangenome landscape of Bacillus: insights from 10,000 genomes into taxonomy, functional potential, and biotechnological applications
Source: World J Microbiol Biotechnol. 2026 Apr 10;42(4):191. doi: 10.1007/s11274-026-04933-x (PMC13068714; doi:10.1007/s11274-026-04933-x)
Supplement: Supplementary file 1 — (PDF 596 KB) [file 11274_2026_4933_MOESM1_ESM.pdf]

Integrative phylogenomic and pangenome landscape of *Bacillus*: insights from 10,000 genomes into taxonomy, functional potential, and biotechnological applications

Hector J. Acho-Vasquez[1], Sarah Henaut-Jacobs[1], Thiago M. Venancio[1,*]

[1] Laboratório de Química e Função de Proteínas e Peptídeos, Centro de Biociências e Biotecnologia, Universidade Estadual do Norte Fluminense Darcy Ribeiro, Campos dos Goytacazes, Brazil. * Correspondent author: tmvenancio@uenf.br.

Supplementary Figures

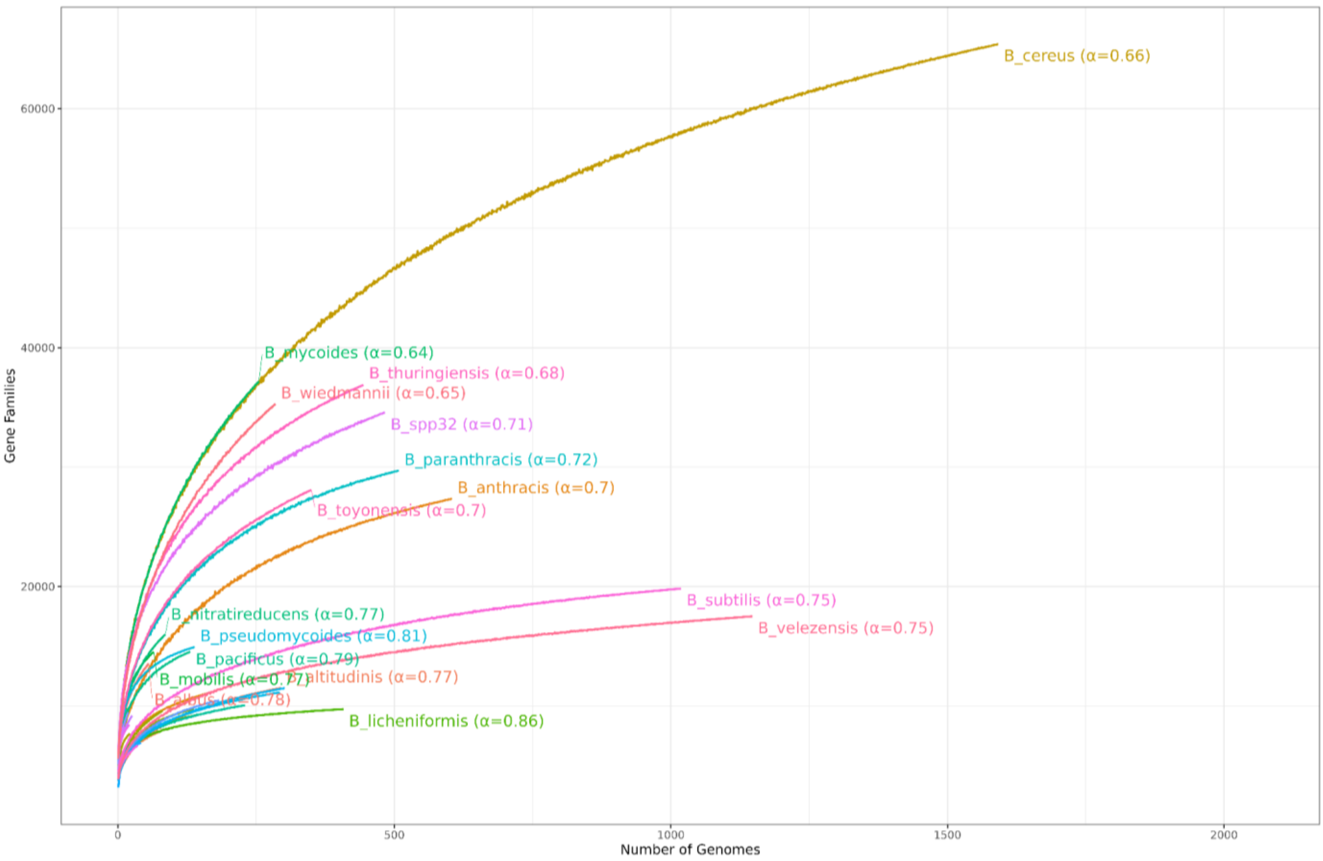

Figure S1. Global *Bacillus* pangenome curves showing gene family accumulation as a function of genome sampling. Each curve represents a distinct Bacillus lineage, with α values indicating the pangenome openness estimated from Heaps' law. Lower α values denote more open pangenomes (greater gene diversity), whereas higher α values indicate more closed pangenomes.

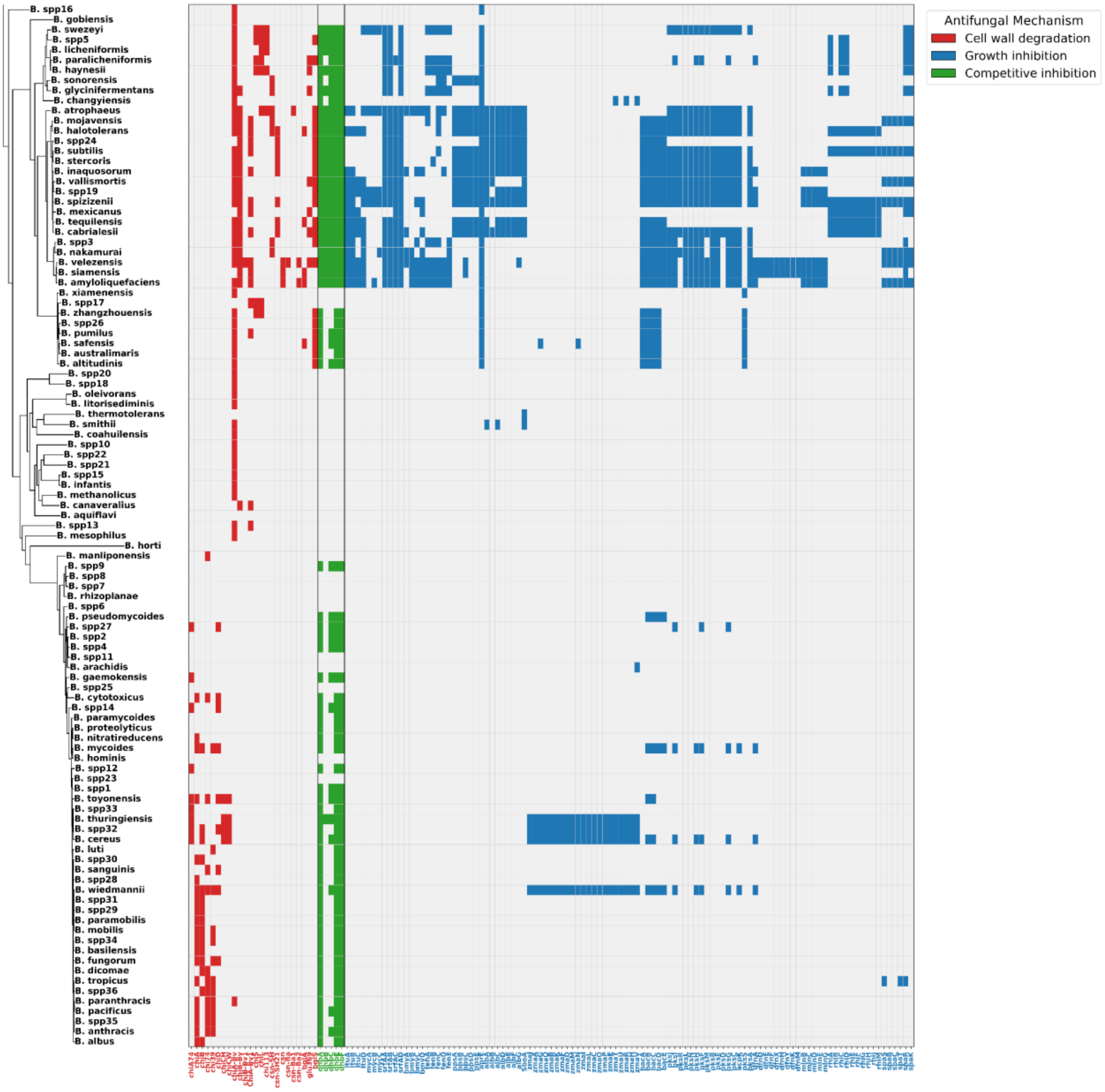

Figure S2. Presence-absence matrix of genes with antifungal potential across *Bacillus* identified using USEARCH v11.0.667. Clusters of genes related to secondary metabolite biosynthesis (e.g., *ituA*, *ituB*, *ituC*, *ituD*) and siderophore production (e.g., *dhbA*, *dhbB*, *dhbC*) are highlighted. The analysis also includes lytic enzyme genes such as chitinases (e.g., *chiA*, *chiL*), chitosanase (e.g., *csn*), and β-glucanases (e.g., *bglA*, *bglS*).

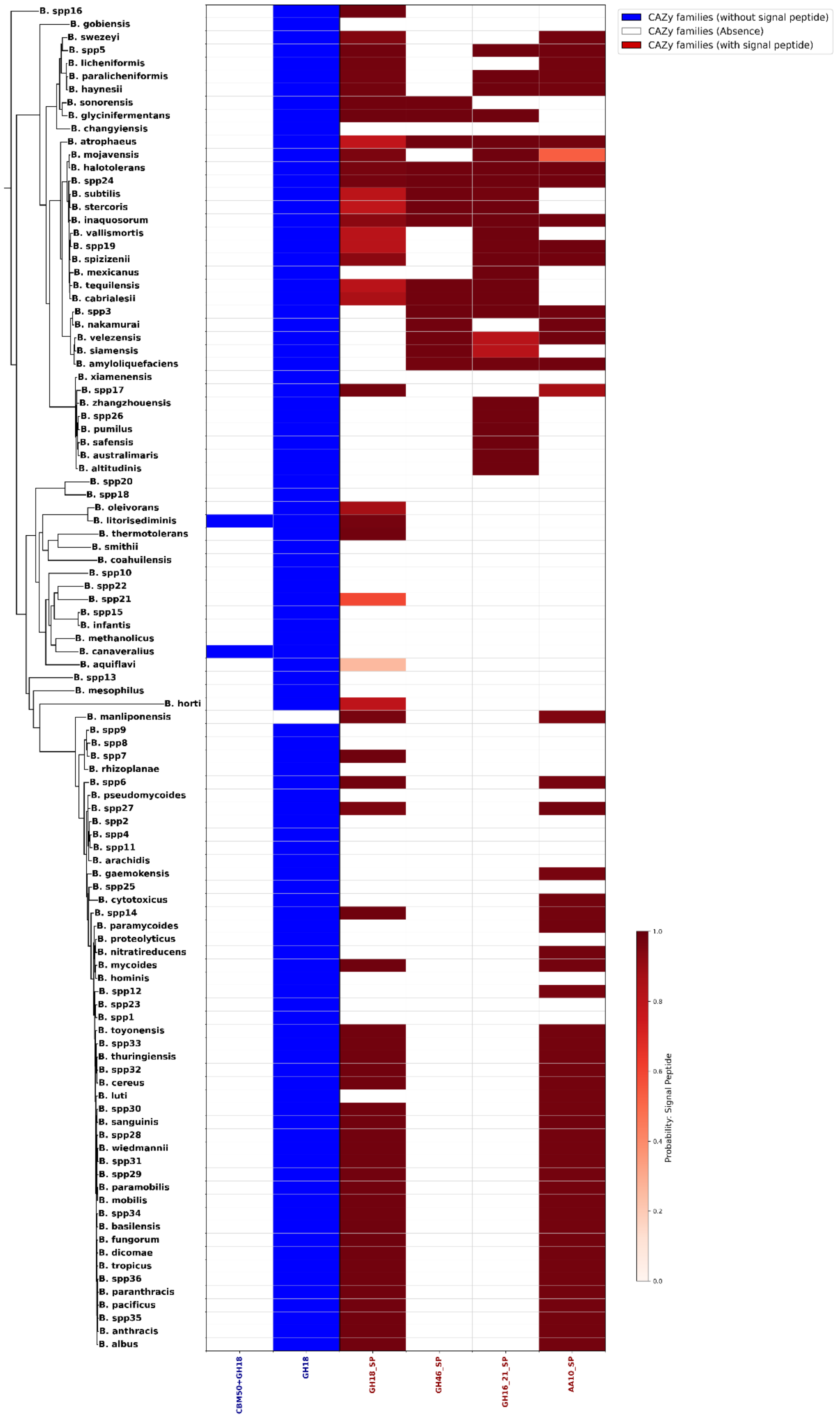

Figure S3. Presence–absence patterns of lytic CAZy families across the *Bacillus* genus. Families lacking signal peptides (e.g., GH18) are shown in blue, whereas signal peptide–bearing families (e.g., GH18_SP and GH46_SP) are shown in red.
